# PD-L1 Inhibitors: Different Classes, Activities, and Mechanisms of Action

**DOI:** 10.3390/ijms222111797

**Published:** 2021-10-30

**Authors:** Ewa Surmiak, Katarzyna Magiera-Mularz, Bogdan Musielak, Damian Muszak, Justyna Kocik-Krol, Radoslaw Kitel, Jacek Plewka, Tad A. Holak, Lukasz Skalniak

**Affiliations:** Department of Organic Chemistry, Faculty of Chemistry, Jagiellonian University, Gronostajowa 2, 30-387 Krakow, Poland; ewa.surmiak@uj.edu.pl (E.S.); k.magiera@uj.edu.pl (K.M.-M.); bogdan.musielak@uj.edu.pl (B.M.); damian.muszak@uj.edu.pl (D.M.); justyna.kocik@doctoral.uj.edu.pl (J.K.-K.); radoslaw.kitel@uj.edu.pl (R.K.); jacek.plewka@uj.edu.pl (J.P.); holak@chemia.uj.edu.pl (T.A.H.)

**Keywords:** PD-L1 inhibitor, immune checkpoint blockade, immunotherapy

## Abstract

Targeting the programmed cell death protein 1/programmed cell death 1 ligand 1 (PD-1/PD-L1) interaction has become an established strategy for cancer immunotherapy. Although hundreds of small-molecule, peptide, and peptidomimetic inhibitors have been proposed in recent years, only a limited number of drug candidates show good PD-1/PD-L1 blocking activity in cell-based assays. In this article, we compare representative molecules from different classes in terms of their PD-1/PD-L1 dissociation capacity measured by HTRF and in vitro bioactivity determined by the immune checkpoint blockade (ICB) co-culture assay. We point to recent discoveries that underscore important differences in the mechanisms of action of these molecules and also indicate one principal feature that needs to be considered, which is the eventual human PD-L1 specificity.

## 1. Introduction

Multiple studies have shown a central role of immune checkpoint molecules (ICMs) in the immune evasion of cancer cells. The blockade of inhibitory ICMs has been shown to restore the activity of T cells, leading to the durable recovery of a significant subset of cancer patients. The therapeutic potential of immune checkpoint blockade was first presented for the CTLA-4/CD80/86 immune checkpoint with ipilimumab (Yervoy), a first-in-class ICM-blocking antibody that was approved in 2011 for the treatment of late-stage melanoma [1]. Following the success of anti-CTLA-4 therapy, anti-tumor immunotherapy has been developed to target the programmed cell death protein 1/programmed cell death 1 ligand 1 (PD-1/PD-L1) interaction. By now, seven therapeutic antibodies targeting either PD-1 or PD-L1 have been approved by the FDA for the treatment of human cancers [2]. The list of approvals is constantly expanding towards more and more cancer types and therapeutic antibodies, which reflects the interest in PD-1/PD-L1 blockade and its prospects for clinical use (Cancer Research Institute, FDA Approval Timeline of Active Immunotherapies, www.cancerresearch.org, 3 October 2021). In parallel, considerable effort has been made in a search for small-molecule drug candidates, which often possess characteristics that can be considered alternative, if not superior, to antibodies.

While targeting PD-1 with small molecules is considered much more difficult to achieve, researchers have focused on designing small molecules that could bind and block PD-L1. Pioneering work in this field was carried out by scientists from Bristol Myers Squibb (BMS), resulting in several patents involving a new biphenyl-based chemical scaffold postulated to block the PD-1/PD-L1 checkpoint [3,4].

Early experimental reports on the structural features of the interaction of biphenyl BMS molecules with human PD-L1 allowed us to further rationalize the structure-activity relationship (SAR) analysis [5,6,7]. Since that time, besides a few exceptions, studies on small molecules targeted at PD-L1 have mainly focused on the SAR analysis of the biphenyl core. Extensive work on biphenyl molecules has resulted in a huge number of publications and patents appearing in the last 5 years [8,9]. However, only a handful of these molecules have progressed to clinical trials [2]. The second group of PD-L1 targeting non-antibody agents consists of macrocyclic peptides, with several notable examples disclosed by Bristol Myers Squibb [10,11]. Peptidomimetics that interfere with the PD-1/PD-L1 immune checkpoint have also been proposed as an alternative to small-molecule and peptide compounds [9].

In addition to the different chemical natures and pharmacological properties of antibodies, small molecules, macrocyclic peptides, and peptidomimetics directed against the PD-1/PD-L1 immune checkpoint, these also exhibit different mechanisms of action and activities. The purpose of this report is to compare the in vitro activities of molecules belonging to different classes and to highlight the functional aspects that should be considered when proceeding with further preclinical and clinical trials.

## 2. Results

### 2.1. The Comparison of In Vitro Activities

**Figure 1:**
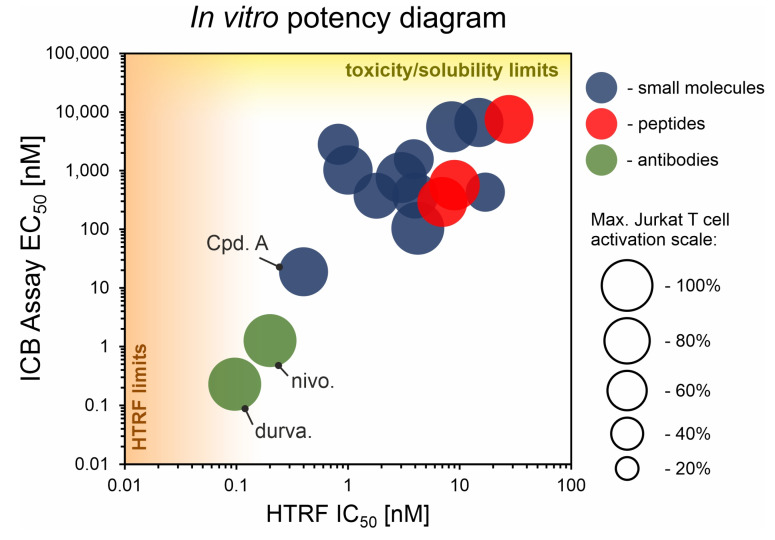
The correlation of IC_50_ values from the HTRF assay and EC_50_ values from the ICB assay for representative molecules belonging to various classes: blue—small molecules; red—peptides; green—antibodies. Each bubble represents a separate PD-L1-targeted molecule listed in Table 1. The size of the bubble indicates the % of the maximal activation of effector Jurkat T cell in the ICB assay, achieved for a therapeutic anti-PD-L1 antibody. “HTRF limit” indicates a bottom limit of IC_50_ determination with HTRF (related to the concentrations of targeted PD-1 and PD-L1 proteins). “Toxicity/solubility limits” indicate the upper limits of EC_50_ determination in the ICB assay (related to toxicity towards the cells and limited water solubility of the molecules). See Appendix A for further explanations. Durva., durvalumab; nivo., nivolumab; Cpd. A, compound A.

We performed a comparison of the in vitro targeting of the PD-1/PD-L1 immune checkpoint with molecules selected from various classes, including small molecules, macrocyclic peptides, and monoclonal antibodies. For this, two standardized and popular techniques used in the available literature were applied: the protein-based Homogeneous Time-Resolved Fluorescence (HTRF) method and the cell-based Immune Checkpoint Blockade (ICB) assay. Using these methods, three parameters describing the in vitro potency of the tested compounds were determined: (i) IC_50_ values of the blockade of PD-1/PD-L1 complex formation (from HTRF); (ii) EC_50_ values of the reactivation of the effector T cells blocked with PD-L1 (from ICB); and (iii) maximal activation levels of the effector T cells, calculated as the % of the activation in the presence of therapeutic anti-PD-L1 antibody atezolizumab or durvalumab. Based on these parameters, a bubble plot was prepared to visualize the differences between groups of molecules (Figure 1). The numeric data are also presented in Table 1. The molecules represent biphenyls [3,4,5,12,13,14,15,16], terphenyls [17,18], biphenyls with a ring fusion [19], elongated biphenyls [20], symmetric biphenyls [21,22,23], and macrocyclic peptides [10,11,24,25].

**Figure 2:**
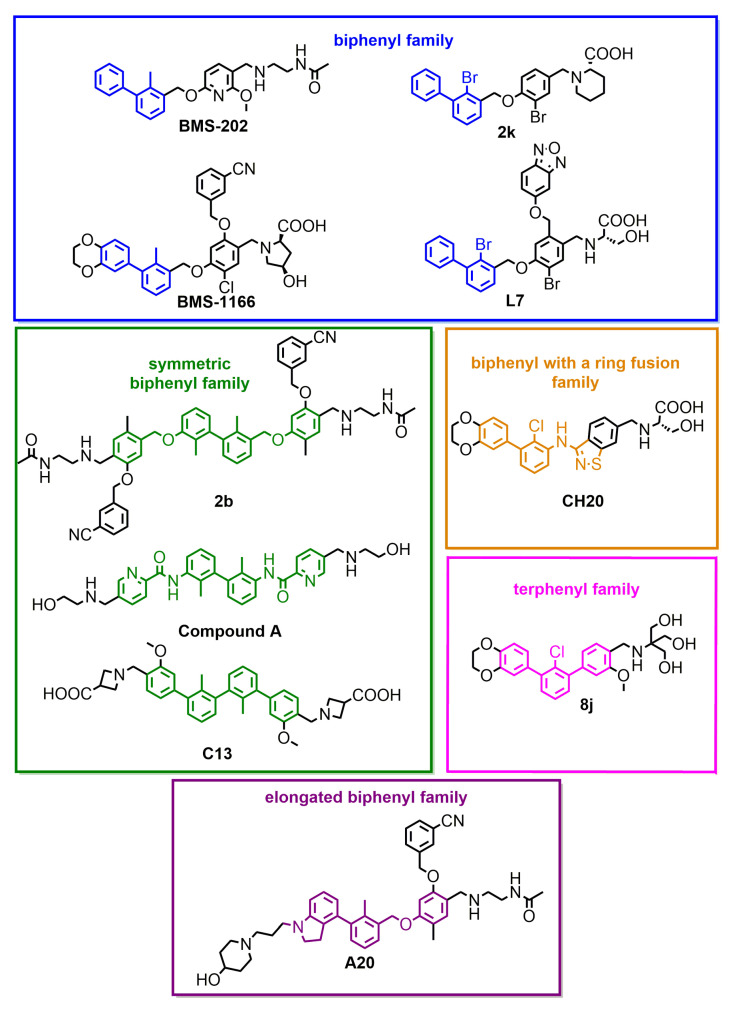
Small-molecule inhibitors of PD-L1, representative of different subclasses of the biphenyl superfamily.

From the graph, it is clear that the therapeutic antibodies, represented by durvalumab and nivolumab, present extraordinary properties, both in the HTRF and ICB assays. In contrast, most of the tested biphenyl-cored small molecules (Figure 2) present a relatively low activity in a cell-based ICB assay, despite their IC_50_ values close to 1 nM, as determined with HTRF. Additionally, the maximal activation levels achievable for the molecules are frequently lower than the levels achieved for antibodies, which is due to the limited water solubilities and cytotoxic effects observed for some molecules when used at higher concentrations (above *c.a.* 2 µM) [5,18,22]. Some other molecules were shown to be less toxic, with 50% cell growth inhibition values above 10–30 µM [12,19,20].

One important exception within small molecules is Compound A, disclosed by Arbutus Biopharma Inc. and published recently by Park and coworkers [21]. While most of the SAR work around the biphenyl core seem to fail to produce sufficiently improved drug candidates, this lone example proves the feasibility of the design of molecules with a good in vitro potency that also have promising in vivo activity [21].

Amongst the macrocyclic peptides, the analyzed representatives present low bioactivity, far lower than the exemplary activities of therapeutic antibodies. The peptides seemed to perform worse in the HTRF yet better in the ICB assay compared to small molecules. For small molecules, it is easier to achieve lower IC_50_ values, yet this does not translate well into a potency in the cellular environment. In this respect, biologics, such as antibodies or macrocyclic peptides, seem to be more suitable when confronted with a target in a biological context.

### 2.2. The Mechanisms of PD-1/PD-L1 Blockade

**Figure 3:**
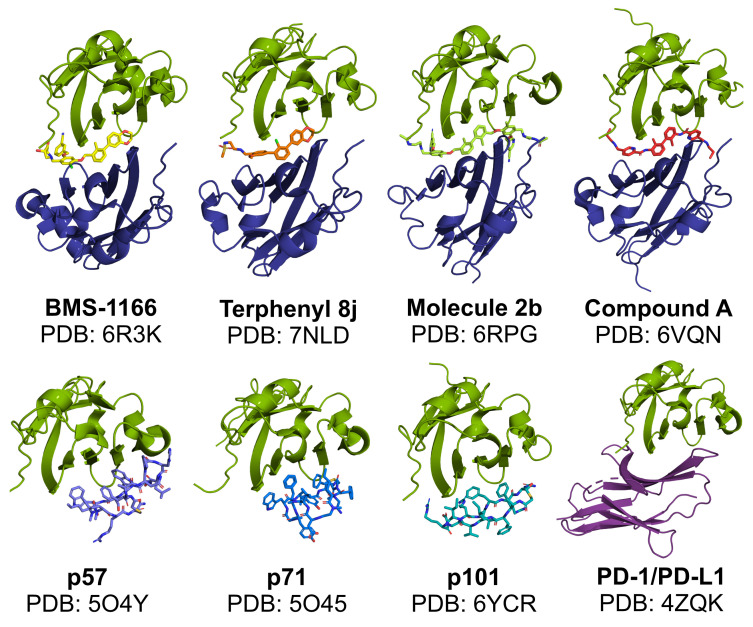
Modes of in vitro binding of PD-L1-targeted molecules to PD-L1. Like therapeutic antibodies, macrocyclic peptides bind and block PD-L1 at a similar surface as recognized by the PD-1 protein (the bottom panel). In contrast, in the presence of biphenyl molecules, a formation of PD-L1 dimers is favored, with a single molecule bound within the interface of the two PD-L1 protomers (the upper panel). Green—_A_PD-L1 protomer; dark blue—_B_PD-L1 protomer; purple—PD-1; other colors: small molecules and macrocyclic peptides.

**Figure 4:**
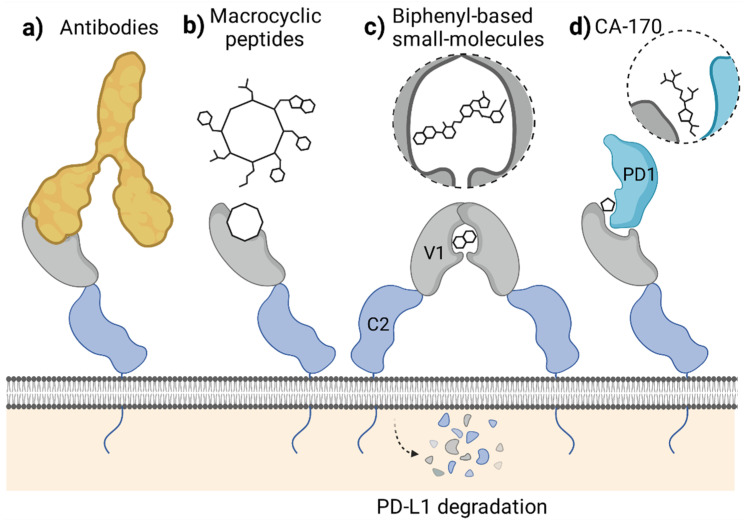
Mechanisms of PD-1/PD-L1 blockade attributed to different classes of molecules targeting the extracellular domain of PD-L1. (**a**,**b**) The blockade of PD-L1 surface with antibodies (**a**) or macrocyclic peptides (**b**) which antagonize PD-1 binding. (**c**) Small-molecule-induced PD-L1 dimerization leading to cell surface PD-L1 loss by the protein maturation blockade or internalization. (**d**) Forming a defective ternary complex between CA-170, PD-1, and PD-L1. Figure created with BioRender.

In classical terms, the immune checkpoint blockade refers to the binding of a molecule to the targeted immunoreceptor and acting as its antagonist, thus preventing the binding of a natural ligand. This disallows checkpoint formation and its physiological functioning. Such a mechanism is observed for the blocking antibodies, including anti-PD-1 and anti-PD-L1 therapeutic antibodies, which effectively compete for binding with either PD-L1 or PD-1 protein. In our studies, the macrocyclic peptide inhibitors of PD-L1 follow this mechanism as they bind to PD-L1 within its large PD-1-binding surface and prevent PD-1 from binding to PD-L1. As such, macrocyclic peptides bind PD-L1 in a 1:1 molar ratio (Figure 3). Thus, macrocyclic peptides seem to resemble the mechanism characteristic for the classical ICB (Figure 4a,b).

Unlike antibodies and macrocyclic peptides, most, if not all, of the known PD-L1-targeted small molecules do not simply block the PD-L1 surface. Instead, the molecules provoke dimerization of the human PD-L1 in vitro, as we reported back in 2016 and 2017 [5,6,7] (Figure 3). This dimerization likely results from the increased hydrophobicity of the already partially hydrophobic surface of PD-L1 upon binding of a small molecule, causing the recruitment of a second hydrophobic surface of another PD-L1 protein. Such a binding model was postulated by us in our previous work based on molecular docking experiments [5] and later confirmed by others [26].

The molecule-induced PD-L1 dimerization was also recently reported by Park and co-workers, who showed the dimerization of PD-L1 in a presence of their symmetric compound A in a cellular context [21]. The authors postulated that this dimerization results in a downregulation of the cell surface expression of PD-L1 in tumor cells [21]. Simultaneously, another group reported that the targeting of PD-L1 with a biphenyl molecule BMS-1166 occurs at the early stages of the protein maturation in the endoplasmic reticulum (ER), preventing its transport into the Golgi apparatus [27]. This, in turn, leads to the under-glycosylation of PD-L1 and its elimination, likely through the ER-associated protein degradation (ERAD) pathway, as evidenced for the constitutive, IFN-γ-induced, and overexpressed human PD-L1, but not mouse PD-L1 [27]. The data concerning the importance of the regulation of PD-L1 maturation, trafficking, and stability are increasing [28]. In fact, several examples of antibody-based molecules designed to target PD-L1 to degradation, such as the LYTACs [29] and AbTACs [30], were proposed. Now, it also seems that small molecules can disable PD-L1 by decreasing its cell-surface content, even though they were initially not intended to do so (Figure 4c).

A third, alternative mode of action of the molecule designed to bind to the extracellular domain of PD-L1 was proposed with the discovery of a peptidomimetic compound CA-170. This molecule has gained special attention in the past few years, as it was the first orally bioavailable small-molecule PD-L1 and VISTA antagonist to enter clinical trials [31]. Initially, CA-170 was intended to block the interaction of PD-L1 and PD-1, as its structure was derived from the peptide sequence of the PD-L1-interacting region of human PD-1. Recently, however, CA-170 was shown to be a direct binder of neither human PD-L1 nor PD-1 and was unable to dissociate the PD-1/PD-L1 complex in various assays [32,33,34]. In response, an alternative mechanism of action of CA-170 was proposed by its inventors, in which the compound would bind to the already formed complex of PD-1 and PD-L1 proteins, creating the “defective ternary complex” and thus disabling this immune checkpoint [35] (Figure 4d). This is a unique alternative theory that attempts to explain CA-170 bioactivity towards PD-1/PD-L1 despite the lack of its binding to any of the checkpoint components alone. While CA-170 was shown to increase the activation of human PBMCs and mouse T cells, the molecule was inactive in a PD-1/PD-L1-focused cell-based ICB assay [34], pointing out that alternative PD-1/PD-L1-independent T cell stimulatory effects cannot be excluded.

### 2.3. Specificity Towards the Human and Mouse PD-L1

When defining the right model for the in vivo evaluation of the bioactivity of a drug candidate, it is crucial to determine interspecies specificity towards the molecular target. We have shown recently that despite the remarkable similarity in the structure and sequence, the mouse and human PD-L1 analogs differ greatly in their druggability profiles [36]. In the case of molecules blocking PD-L1 protein, it is thus crucial, but rare, in the manuscripts to verify the interaction with mouse PD-L1 before proceeding to the immunocompetent syngeneic mouse models. As an alternative, humanized knock-in animals can be used to avoid interspecies specificity complications with PD-L1 binding.

Out of the tested therapeutic antibodies, atezolizumab blocks both human and mouse PD-L1 (*m*PD-L1). Durvalumab blocks the human PD-L1 but not the mouse PD-L1 protein, as we have shown in our recent manuscript [36]. In our hands, none of the small molecules and macrocyclic peptides tested in our laboratory were able to bind to mouse PD-L1, as we have verified either with the NMR study or the hybrid, *m*PD-L1/*h*PD-1 ICB assay [36]. Importantly, similar observations have been reported by others. In their conference abstracts, researchers from ChemoCentryx reported the discovery of small-molecule inhibitors of PD-1/PD-L1 that are human-specific [37]. Therefore, in subsequent studies, a humanized MC38-*h*PD-L1 tumor model was used [38]. Similar cells engrafted into humanized double knock-in mice for humanized PD-1 and PD-L1 (C57BL/6- Pdcd1tm1(PDCD1) Cd274tm1(CD274)/Bcgen) were used by Park and co-workers, who succeeded in showing the bioactivity of their symmetric drug candidate, depicted as Compound A [21]. Similar humanized animals were also used by others to verify the bioactivity of the aliphatic amine-linked triaryl derivatives of biphenyl molecules [16].

On the other hand, some groups have defined the interaction of their molecules with human and mouse PD-L1 before deciding on using the mouse model for their study. Wang and coworkers reported that optimized biphenyl pyridine compound binds to the mouse PD-L1 significantly more weakly than to the human PD-L1, as calculated from the microscale thermophoresis (MST) assay (K_D_ of 45 nM for *h*PD-L1 and 2.3 µM for *m*PD-L1) [39]. Despite the difference in binding constants, a classical mouse model was used for the study of in vivo bioactivity of the compound. In another study, resorcinol derivatives of biphenyls were shown to bind to the mouse and human PD-L1 with comparable affinities [15]. Such a characteristic seem to justify the use of non-humanized, classical, immunocompetent mouse models. Yet, for some reason, in the study humanized PD-1 animals were used, although in combination with non-humanized cell lines [15,40].

All the above-mentioned concerns highlight the requirement for the transparent analysis of binding of the molecules not only to the human but also to the mouse PD-L1 to fully justify the choice of animal model used. This will allow us to minimize the risk of unspecific, off-target effects being evoked instead of the intended targeting of the PD-1/PD-L1 immune checkpoint.

## 3. Discussion

In recent years, extensive work has been conducted in the search for novel non-antibody molecules able to block PD-L1. This has led to the discovery of hundreds of small molecules, peptides, and peptidomimetics disclosed in numerous patents and published in numerous articles. Most of this work features the SAR analysis of biphenyl-cored molecules discovered by Bristol Myers Squibb. Although some new ideas such as symmetric molecules [21,22], terphenyls [17,18], or indolines [41] were proposed, only a moderate improvement of the bioactivity of the molecules could be achieved. It seems that the structure optimization of small molecules relying only on protein-based assays, such as the HTRF, is insufficient since many compounds present IC_50_ values close to the lower limit of determination, but without a clear translation to the biological activity in a more complex, cellular environment. Only a few examples, such as compound A [21], seem to break the wall of limited bioactivity and present promising potency in a cell-based ICB assay. In the case of macrocyclic peptides, similar bioactivities as for small molecules are observed, even despite higher IC_50_ values in HTRF assay. Still, the activities of these drug candidates are far lower than the activities of the therapeutic antibodies.

Notably, the different classes of PD-L1 inhibitors present various mechanisms of action—i.e., a classical PD-L1 blockade, PD-L1 internalization, blockade of PD-L1 maturation, or putative formation of a defective ternary complex. It is possible that focusing primarily on these unique characteristic mechanisms instead of a struggle for better affinities would bring additional improvement in the development of PD-L1 inhibitors. Another crucial aspect, which needs to be considered when progressing towards the in vivo setups, is the ability of the molecules to target the mouse PD-L1. Since for many molecules, human PD-L1 specificity was presented, it is of the highest importance to choose the right in vivo model based on the characteristics of a particular drug candidate. Choosing the classical immunocompetent mouse model without proving the blockade of *m*PD-L1/PD-1 immune checkpoint carries the threat of wasting time and resources on negative results or evoking off-target effects only. Alternatively, human 3D tumor cultures and patient-derived organoids could be considered for the pre-clinical testing of experimental PD-1/PD-L1 immune checkpoint blockade therapies. These constitute more advanced and more physiologically relevant models than artificial setups such as the ICB assay and have the benefit of providing the human molecular targets and human context required for the proper testing of human-dedicated drug candidates [42,43,44,45].

## 4. Materials and Methods

### 4.1. Homogeneous Time-Resolved Fluorescence Assay

The certified CisBio (Codolet, France) HTRF assay was used to determine the IC_50_ values of the compounds according to the manufacturer’s guidelines. The system consists of *h*PD-1, *h*PD-L1, anti-Tag1 labeled with Europium cryptate (which serves as the HTRF donor), and anti-Tag2 labeled with XL665 (which works as the HTRF acceptor). The experiments were performed in 20 μL final volume using 5 nM *h*PD-L1 and 50 nM *h*PD-1 in their final formulation according to the affinities determined by the manufacturers for this particular assay. IC_50_ of tested compounds was determined for two individual dilution series unless stated otherwise. After mixing, the plate was left for 2 h incubation at room temperature and later measured using Tecan Spark 20M multimode microplate reader (Tecan, Männedorf, Switzerland). The observed fluorescence comes when the donor and acceptor antibodies are within proximity (so-called FRET distance) due to PD-L1 and PD-1 interacting. By excitation of the donor fluorophore, the fluorescence resonance energy transfer (FRET) is triggered towards the acceptor fluorophore emitting the fluorescence at 665 nm proportional to the extent of PD1/PD-L1 interaction. Therefore, compounds blocking PD-1/PD-L1 interactions will reduce the observed HTRF signal. Collected data were background-subtracted on the negative control (no PD-1—complex cannot be created) and normalized on the positive control (the volume of the compound is replaced by buffer—full complex signal). After, averaging data were fitted using the Mathematica 12 software with the normalized Hill’s equation to determine the IC_50_ value.

### 4.2. PD-1/PD-L1 ICB Assay

The immune checkpoint blockade (ICB) of the PD-1/PD-L1 interaction was performed with the use of the PD-1/PD-L1 Blockade Bioassay (Promega, Madison, WI, USA) according to the manufacturer’s protocol.

In this assay, two cell lines are co-cultured: artificial antigen-presenting CHO-K1 cells overexpressing PD-L1 and a TCR Activator protein (CHO/TCRAct/PD-L1 cells), and Jurkat T cells overexpressing PD-1 and luciferase gene controlled by NFAT Response Element (Jurkat Effector Cells, Jurkat- ECs). The binding of TCR Activator with TCR leads to the activation of Jurkat-ECs, involving the activation of NFAT transcription factor. NFAT induces the expression of luciferase, which provides the readout in the experiment. At the same time, PD-1/PD-L1 interaction provides a functional immune checkpoint that diminishes this activation. Upon the blockade of the PD-1/PD-L1 checkpoint, the maximal level of Jurkat-ECs activation is restored. This allows the evaluation of PD-1/PD-L1-targeting molecules in a cellular context.

CHO/TCRAct/PD-L1 cells were seeded on 96-well white plates at the density of 10,000 cells/well and the next day the effector PD-1 Jurkat T cells were added (20,000 cells/well) in the presence of increasing concentrations of the compounds with DMSO as a control (the concentration of DMSO was kept constant at 0.1%). The activation of the effector cells was monitored by luminescence measurements after 6 h of incubation (37 °C, 5% CO_2_) and 20 min of additional incubation with the Bio-Glo assay reagent (Promega) at room temperature. The luminescence was detected with the Tecan Spark 20M multimode microplate reader (Tecan). The data are presented as the fold induction of the luminescence signal relative to DMSO-treated cells. Data points represent mean ± SD values from three independent experiments. Half-maximal effective concentrations (EC_50_ values) were calculated from the Hill curve fitting to the experimental data using the Origin Pro 2020 software (OriginLab Corporation, Northampton, MA, USA).

## 5. Conclusions

In summary, although various classes of PD-L1 inhibitors have been proposed in the last few years, the properties of non-antibody molecules need to be improved and verified with the use of proper models if they are to compete with approved antibody-based therapies.

## Figures and Tables

**Table 1:** The list of representative PD-L1 inhibitors belonging to various classes and their PD-L1 blockade characteristics. IC_50_ values were determined with the HTRF assay and EC_50_ values were determined with the ICB assay. %RLU_max_ indicates the maximal activation of Jurkat T cells in the ICB assay, calculated as the % of the activation achieved for therapeutic anti-PD-L1 antibody (atezolizumab or durvalumab). When available, the data on the in vitro PD-L1 dimerization in the presence of the molecule, and the species specificity (human PD-L1, *h*PD-L1, and mouse PD-L1, *m*PD-L1) are indicated. “√”, particular activity confirmed experimentally; “No”, the compound was confirmed not to possess a particular activity; “n.d.”, no data on a particular activity.

Class	Name	HTRF IC_50_ [nM]		ICB Assay			PD-L1 Dimerization	Target Specificity	
		Other	Ours	EC_50_ [nM]	%RLU_max_	Ref.		*h*PD-L1	*m*PD-L1
**Small molecules**	BMS-202	18 [3]	96 [22]	no act.			√	√	No
	BMS-1166	1.4 [4]	3.89 [18]	1574	47	(Figure A1)	√	√	No
	2k		14.9 [12]	6632	87	[12]	√	√	No
	8j		<1 [18]	1026	87	[18]	√	√	No
	A20	17 [20]		430	55	[20]	n.d.	√	n.d.
	CH20	8.5 [19]		5600	93	[19]	n.d.	√	n.d.
	L7	1.8 [13]		375	75	[13]	n.d.	√	n.d.
	C13	4.23 [23]		104	100	[23]	n.d.	√	n.d.
	2b		3 [22]	763	93	[22]	√	√	No
	comp. A	0.4 [21]		18.9	86.3	[21]	√	√	No
**Macrocyclic peptides**	p57	9 [10]		566	91	[25]	No	√	No
	p71	7 [10]		293	89	[25]	No	√	No
	p99	153 [10]		6300	83	[25]	No	√	No
	p101	120 [10]	27.75 [24]	7500	85	[24]	No	√	No
**Antibodies**	atezolizumab			0.14	100	[18]	No	√	√
	durvalumab		0.1 (Figure A2)	0.23	100	[24]	No	√	No
	nivolumab	0.2 [21]		1.27	100	[25]	-	-	-
	MIH1							√	No
	MIH5							No	√
	*h*PD-1							√	√

**Figure A1:**
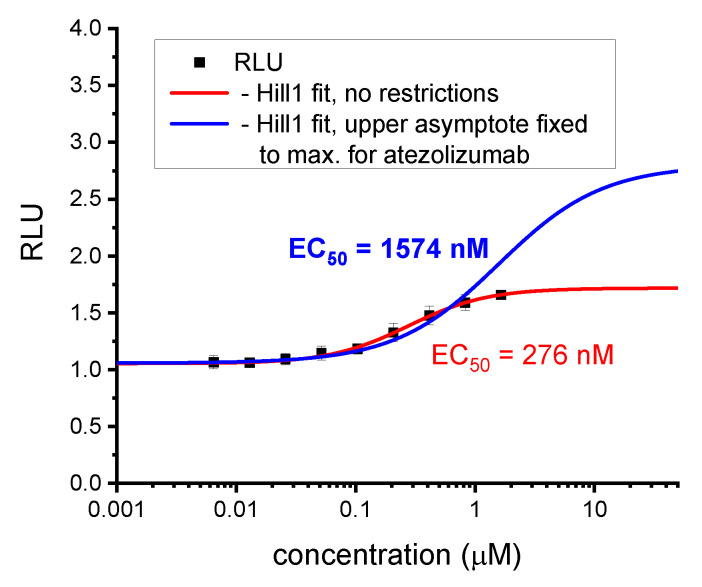
**Re-calculation of the EC_50_ value for BMS-1166 according to the optimized method.** Initial calculations for the biphenyl BMS molecules were carried out without any restrictions to the fitting parameters [5]. With time, we learned that this may lead to depreciated EC_50_ values, since the upper plateau of the experimental points, and thus the upper asymptote of the fitted curve may be lowered by unspecific effects such as cell toxicity. Therefore, in subsequent analyses, we fixed the upper asymptote of curve fittings at a value observed for therapeutic anti-PD-L1 antibody, either atezolizumab or durvalumab. These values were considered maximal effector Jurkat T cells activation levels achievable in the assay. As shown in this figure, such an analysis leads to determining higher EC_50_ values, which in our opinion are more relevant and better reflect the real bioactivity in the ICB assay.

**Figure A2:**
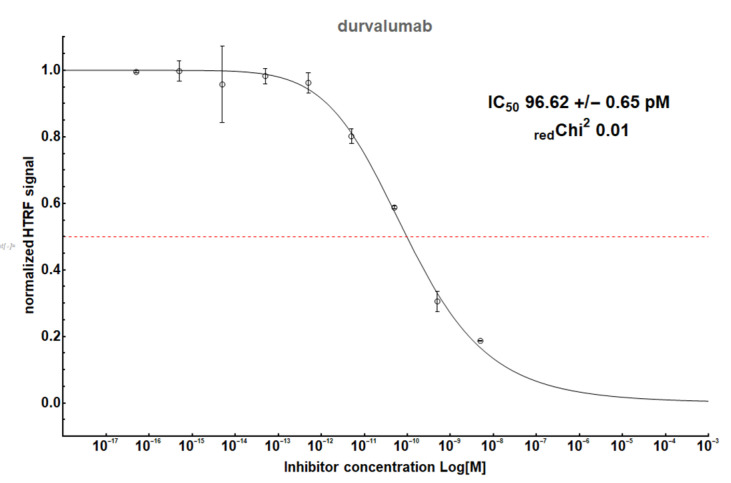
The HTRF analysis result for a therapeutic antibody, durvalumab.

## Data Availability

The data presented in this study include data available in RCSB Protein Data Bank (RCSB PDB), reference numbers: 6R3K, 7NLD, 6RPG, 6VQN, 5O4Y, 5O45, 6YCR, and 4ZQK.

## Appendix A

### HTRF Limitations

HTRF is an extremely popular technique for the initial screening of putative hits against PD-1/PD-L1 systems. as it readily provides the user with the inhibitory potency of the compound represented as the half-maximal inhibitory concentration (IC_50_). IC_50_ indicates how much of a tested substance is required to achieve 50% of the inhibition of a given biological process under specific conditions. Despite being a golden standard for both academia and the pharmaceutical industry since the 1950s, IC_50_ determination heavily depends on both the target protein and ligand concentration as well as the process environment; therefore, the comparison of IC_50_ determined in various laboratories often leads to different results. Moreover, IC_50_ does not include any information regarding the mode of the inhibition (tight-binders, competitive, etc.) as it simply models the response of the fluorophore intensity due to the presence of the complex by means of FRET as a function of the gradient of ligand concentration using four parameters Hill’s equation. Because of that, there is a general trend to move towards other ways to determine the affinity of the putative inhibitors, such as apparent Ki or inhibitory constant, which would make the comparison of the data more feasible. However, the change is slow as it usually requires additional experiments. It is worth mentioning that not all inhibitors can be assessed using HTRF. First, since IC_50_ determination depends on the target protein concentration, theoretically values below 1 nM should not be achievable, unless the protein concentration in the assay is lowered accordingly. Moreover, auto fluorophores or fluorescent quenchers may artificially produce “false-positive” or “false-negative” signals by manipulating the fluorescence readouts.

### ICB Limitations and Transition of HTRF Potency to Activity in the ICB Assay

The half-maximal effective concentration (EC_50_) derived from the cell-based assays represents a completely different value as compared to IC_50_. It is calculated not for the isolated protein system such as IC_50_, but usually for whole cells treated with a particular inhibitor. Moreover, in most cases, protein systems in cell-based assays are subjected to other effects that isolated protein systems cannot address, such as post-translational modifications of the receptors including glycosylation and molecular crowding effect. Therefore, it is not surprising that EC_50_ values of potent “hits” selected from the preliminary HTRF screen are 3–5 magnitudes higher than their corresponding IC_50_ values, if active at all. In addition, since (i) in general higher concentrations of the compounds are required for bioactivity in the ICB assay than the HTRF assay, (ii) small-molecule inhibitors of PD-L1 comprise multiple hydrophobic rings and residues, and (iii) the ICB assay is performed in an aqueous solvent (cell culture medium) with limited tolerance to DMSO (usually below 0.5%), solubility problems will likely occur at higher concentrations, along with unspecific toxicity, both of which limit the possibility of EC_50_ determination for weaker molecules.
